# Competitive Real-Time Near Infrared (NIR) Vein Finder Imaging Device to Improve Peripheral Subcutaneous Vein Selection in Venipuncture for Clinical Laboratory Testing

**DOI:** 10.3390/mi12040373

**Published:** 2021-03-30

**Authors:** Mark D. Francisco, Wen-Fan Chen, Cheng-Tang Pan, Ming-Cheng Lin, Zhi-Hong Wen, Chien-Feng Liao, Yow-Ling Shiue

**Affiliations:** 1 Institute of Biomedical Sciences, National Sun Yat-sen University (NSYSU), Kaohsiung 80424, Taiwan; markfrancisco_trinity@mem.nsysu.edu.tw; 2 Department of Mechanical and Electro-Mechanical Engineering, NSYSU, Kaohsiung 80424, Taiwan; pan@mem.nsysu.edu.tw (C.-T.P.); leo1127@mem.nsysu.edu.tw (M.-C.L.); 3 College of Medical Technology, Trinity University of Asia (TUA), Quezon City 1102, Philippines; 4 Institute of Medical Science and Technology, NSYSU, Kaohsiung 80424, Taiwan; sallychen@imst.nsysu.edu.tw; 5 Institute of Precision Medicine, NSYSU, Kaohsiung 80424, Taiwan; 6 Department of Mechanical Engineering, R.O.C. Military Academy, Kaohsiung 83059, Taiwan; 7 Department of Marine Biotechnology and Resources, NSYSU, Kaohsiung 80424, Taiwan; wzh@mail.nsysu.edu.tw; 8 Department of Emergency Medicine, Kaohsiung Armed Forces General Hospital, Kaohsiung City 80284, Taiwan

**Keywords:** (NIR) near-infrared, (LED) light emitting diode, vein finder, deoxyhemoglobin, cannulation, venipuncture, image processing

## Abstract

In this study, near-infrared (NIR) technology was utilized to develop a low-cost real-time near infrared (NIR) guiding device for cannulation. A portable device that can be used by medical practitioners and also by students for their skills development training in performing cannulation. Methods. First, is the development of a reflectance type optical vein finder using three (3) light emitting diode (LED) lights with 960 nm wavelength, complementary metal-oxide-semiconductor-infrared (CMOS-IR) sensor camera with 1920 × 1080 UXGA (1080P), IR filter set for the given wavelength, and an open-source image processing software. Second, is the actual in-vitro human testing in two sites: the arm and dorsal hand of 242 subjects. The following parameters were included, such as gender, age, mass index (BMI), and skin tone. In order to maximize the assessment process towards the device, the researchers included the arm circumference. This augmented subcutaneous vein imaging study using the develop vein finder device compared the difference in the captured vein images through visual and digital imaging approaches. The human testing was performed in accordance with the ethical standards of the Trinity University of Asia—Institutional Ethics Review Committee (TUA—IERC). Results. The NIR imaging system of the developed vein finder in this study showed its capability as an efficient guiding device through real-time vein pattern recognition, for both sites. Improved captured vein images were observed, having 100% visibility of vein patterns on the dorsal hand site. Fourteen (5.79%) out of 242 subjects reported non-visible peripheral subcutaneous veins in the arm sites. Conclusions. The developed vein finder device with the NIR technology and reflected light principle with low-energy consumption was efficient for real-time peripheral subcutaneous vein imaging without the application of a tourniquet. This might be utilized as a guiding device in locating the vein for the purpose of cannulation, at a very low cost as compared to the commercially available vein finders. Moreover, it may be used as an instructional device for student training in performing cannulation.

## 1. Introduction

Through the years, the application of light emitting diode (LED) in the field of medical healthcare is highly utilized, primarily due to its efficiency. Vein finders were developed as guiding device in locating the vein for cannulation. In general, approximately 90–95% of hospital patients receive their treatment through intravenous route (IV) of administration [1]. Moreover, more than one billion venipunctures are being performed annually for medical laboratory testing [2]. The said procedures were performed through cannulation. It is a technique in which a cannula is placed inside a vein to provide venous access for blood sampling, drug, fluids, and nutrition administration [3]. In order to perform this procedure, the first step is to make the target vein visible, usually by using a tourniquet or by slightly tapping the site [4]. However, not all of the veins are visible, because they lack the distinguishing color or venous distention of the skin surface, even after the application of a tourniquet [5].

**Figure 1:**
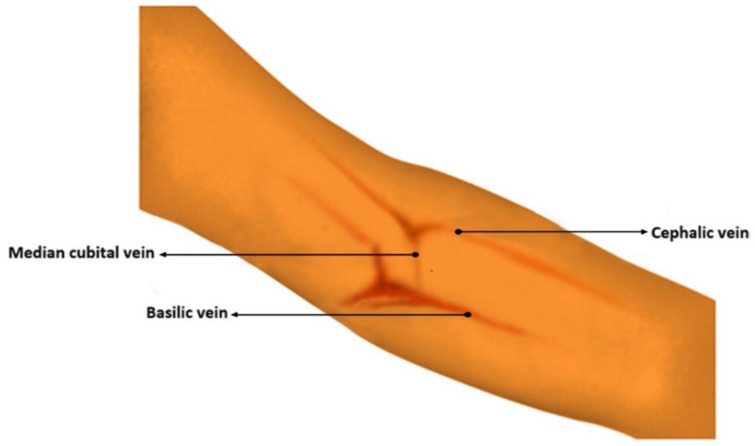
Main veins of the antecubital fossa.

There are three veins that can be seen in the antecubital fossa. Among the three veins at the center of the arm is the median cubital, which should be the first choice, followed by the veins in the lateral aspect (outer thumb side), which is the cephalic vein, and the last are the veins in the medial aspect (inner little finger side), the basilic vein [6]. The median cubital vein is considered as the best site for venipuncture. It serves as a branching between the two other veins [7], described to be well anchored, and usually large and prominent [8]. These were the veins that were observed during the prototype testing with emphasis on the median cubital vein [9]. Figure 1 shows the antecubital fossa with the veins for venipuncture.

Cannulation is considered to be challenging for many clinicians and other medical practitioners to successfully complete it on the first attempt [10], due to the following factors, which causes difficulty during the performance; such are dehydration, dark skin pigmentation, obesity, poor vein quality, aging, and young age, including the low skill of the personnel performing the procedure [11]. This Peripheral Difficult Venous Access (PDVA) is common, and it occurs frequently in clinical settings due to poorly visualized subcutaneous veins [12] causing unsuccessful and prolonged procedure that can lead to patient’s distress, trauma, subcutaneous hemorrhage from frequent attempts, pain, and even unavoidable extreme reactions from children, adults, or patients with mental illness [4,13]. Moreover, failures in this procedure are considered to be the possible sources of variability in the medical laboratory test results. They are parts of the pre-analytical phase errors with the highest percentage of 46.00% up to 77.10%, when compared to the analytical and post-analytical phases [14,15,16,17,18,19,20].

**Figure 2:**
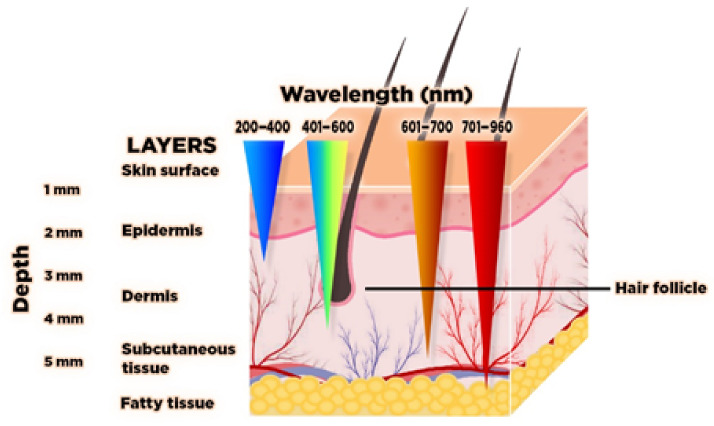
Light skin penetration with different wavelengths (nm).

A vein finder device is primarily composed of a high power NIR-LED, which is the light source, an infrared-sensitive camera, a sensor to capture and format the acquired image real time [21], and a filter for purification by blocking unwanted wavelengths [22,23,24,25,26,27]. To mention, in previous studies, a low-cost LED as light source with complementary metal-oxide-semiconductor (CMOS) imaging sensors was utilized for spectrally encoded confocal microscopy (SECM) as an example of Reflectance confocal microscopy (RCM), a technology for acquiring confocal images [28]. This technology was primarily developed to provide a clearer visible image of the superficial veins. There are two basic principles for vein finders: the reflected light and transillumination. The reflected light type is commonly applied to hand-vein scanners and other commercial devices. The light from the source is reflected in the focused site, and the image is captured by a light sensitive camera at a given wavelength. For transillumination, the light is allowed to penetrate the skin and tissue of the site, and it is then followed by image captured by a camera [27]. The light source can be considered to be the main component of the device. With the electromagnetic spectrum’s range of 740 nm to 940 nm, the light can penetrate to about 5 mm deep of the skin tissue reaching the subcutaneous vein [29] together with the fat cells, arteries, and nerves [30]. Skin penetration of light is with various wavelengths (Figure 2). The lower wavelength of 200–400 nm can only reach the epidermis skin layer, while 400–600 nm up to the dermis skin layer, and 600–700 nm can reach the skin subcutaneous tissue [31,32]. Moreover, a variety of lasers and light sources other than the pulsed dye laser (PDL) are useful in the treatment of vascular lesions: continuous and millisecond pulsed lasers with wavelengths between green and yellow 532 nm and 585–600 nm, near infrared lasers 755-nm, 800–810-nm diode, 1064-nm [33].

Veins are blood vessels that function to transport deoxygenated blood back to the heart [26]. This deoxygenated blood with hemoglobin forms a dark contrast to the skin tissue, because of the absorption of the NIR light making veins more visible [29]. NIR is considered to be a low energy, less reactive wavelength of light widely used for safe clinical use [34].

The development of a low-cost vein finder utilizing the near infrared region (NIR) of the electromagnetic spectrum has become popular. It is to primarily address the concern with the cost of a commercial vein finder with an estimated value of about 4500 USD for portable type to ~27,000 USD for non-portable [35]. Currently, a more convenient model for daily use with optional stand to free the hands of the person performing the cannulation procedure is available at a price range of 4000 USD to 7000 USD per unit [36]. To mention some of the commercialized vein finders are the AccuVein AV300 (AccuVein LLC, Cold Spring Harbor, NY, USA), Vein Viewer (Luminetx, Memphis, TN, USA), [22], VascuLuminator, and the [12]. Previous studies show the capability of vein finders to gain more accurate vein course visualization. Unfortunately, it is not widely utilized, due to the cost of commercially available devices [37].

More so, the objective of the study is to develop an efficient low-cost portable device that can be primarily used as an instructional tool in training students performing cannulation.

It is a non-skin contact vein finder prototype that establishes real-time clear venous images, especially with individuals having non-palpable or visible peripheral subcutaneous vein for fast venous access and success in the first attempt in cannulation. Additionally, to aid in eliminating pre-analytical phase errors in the medical laboratory associated with venipuncture that affects the integrity of laboratory test results.

## 2. Materials and Methods

This section describes the method used in the study. It is composed of two phases: first is the development of a low-cost effective vein finder prototype, and, second, is the actual in-vitro human testing with the device in two different sites, the arm and dorsal hand of 242 volunteers to maximize the assessment process. A tourniquet was not used during the experiment, in order to maintain the non-skin contact technique. The captured images from the device with the Vds Vein display system 4.0 software were used for vein mapping and analysis. The human testing was performed in accordance with the ethical standards of the Trinity University of Asia–Institutional Ethics Review Committee (TUA-IERC), with regard to personal data protection and a subject’s confidentiality.

### 2.1. Device Design with Its Components

**Figure 3:**
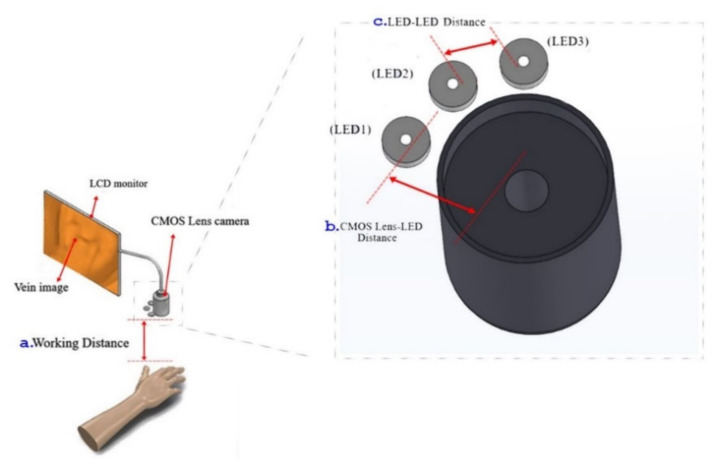
Schematic diagram of the vein finder device design.

**Figure 4:**
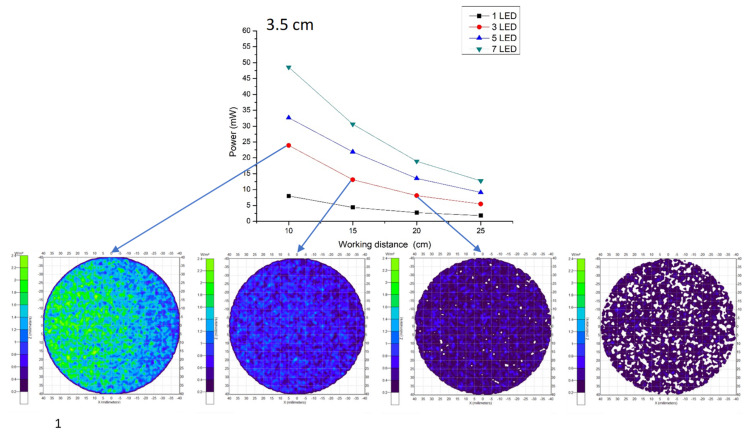
Light emitting diode (LED) power distribution at 3.5 cm CMOS Lens–LED configuration in different working distances.

**Figure 5:**
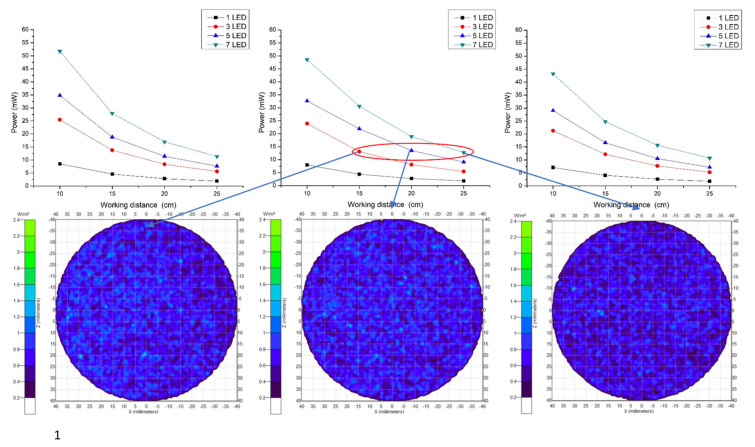
Relationship between working distance and the number of LEDs used.

**Figure 6:**
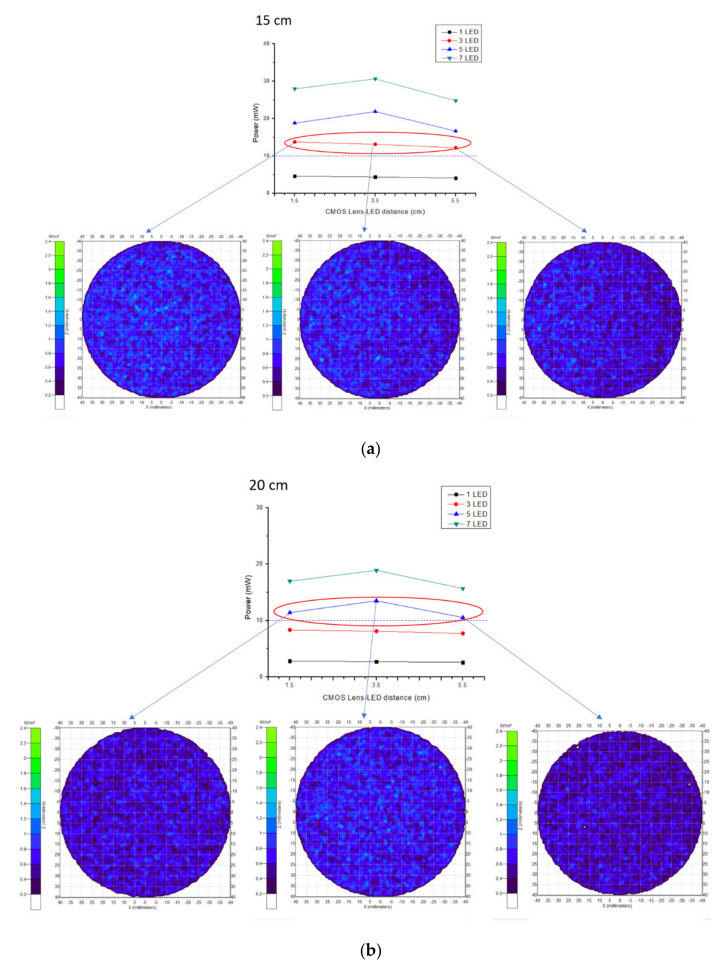

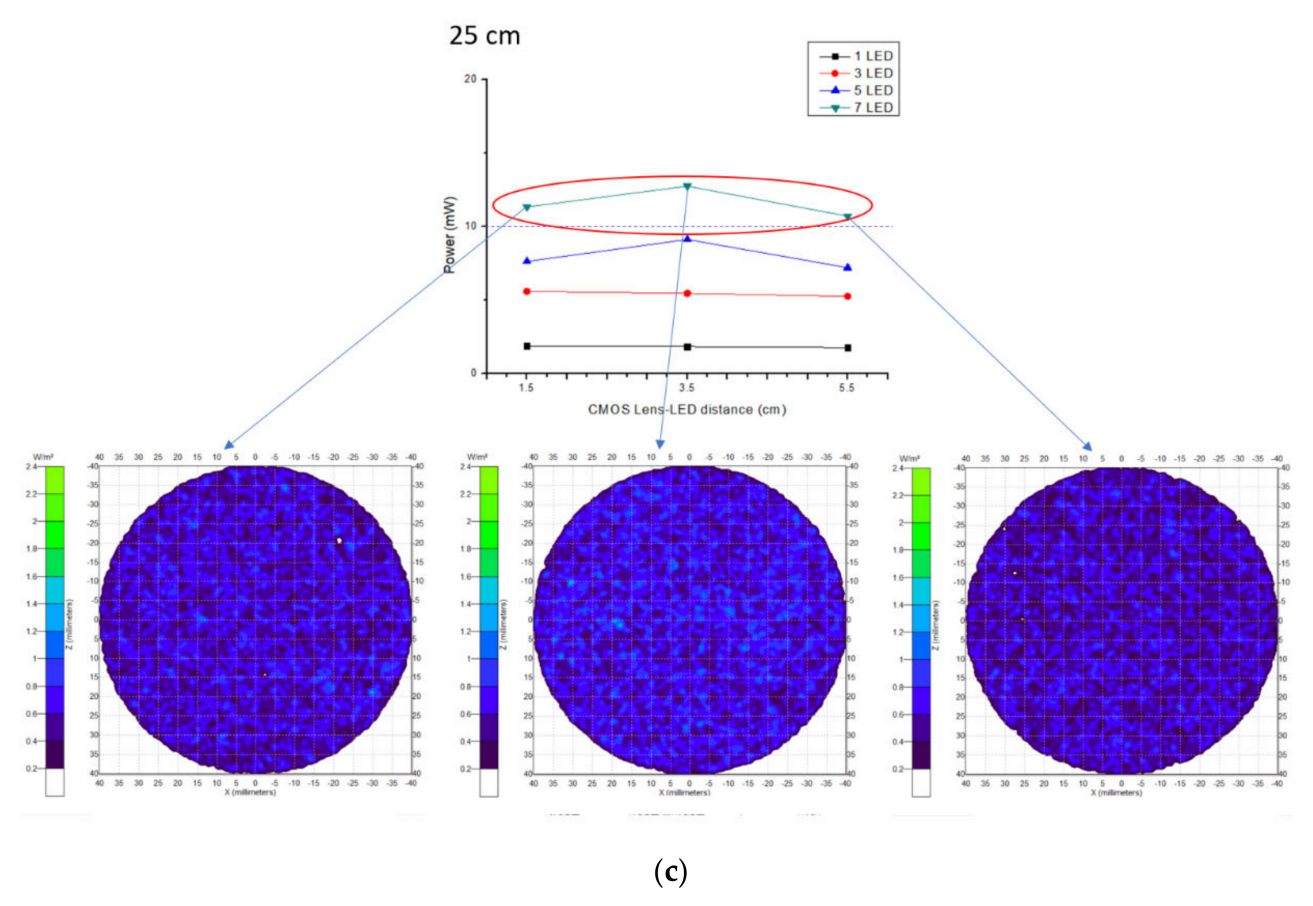
Power strength with different CMOS Lens–LED distances (**a**–**c**).

**Figure 7:**
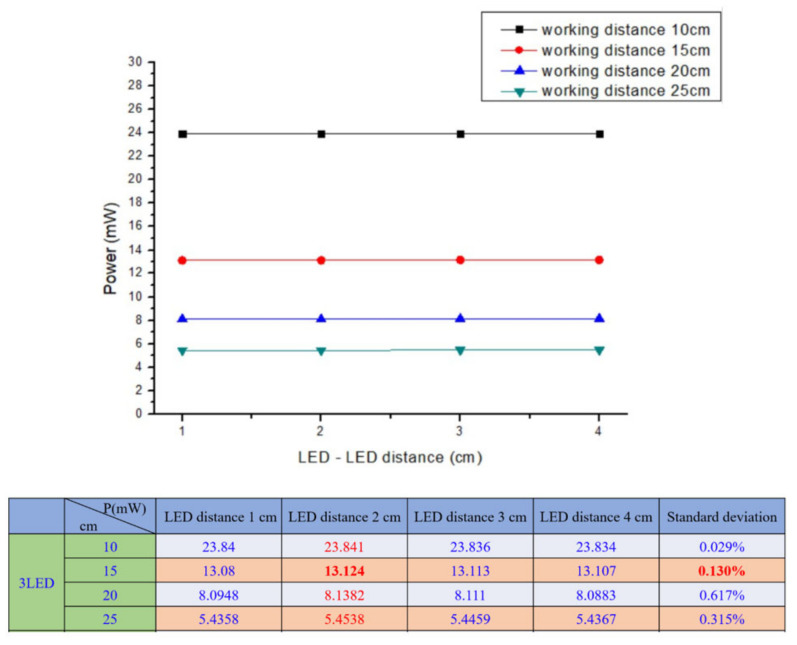
Power distribution in different LED–LED distances.

The performed experiment for wavelength selection showed that near infrared light rays of 960 nm exhibit high absorption with deoxyhemoglobin and deep penetration, achieving the aimed subcutaneous vein visibility. A 1.5 V DC battery powered the three (3) pieces of LED light source. The arrangement of the 3 NIR LEDs is set at a distance of 2.0 cm. It is attached to the camera with 3.5 cm distance from the center, giving the overall diffusion and even radiance. A ½.7″ CMOS based sensor camera with 1920 × 1080 UXGA (1080P) was used. CMOS as IR detector is characterized by high uniformity, low noise, low power consumption, low cost, and high-speed performance/faster readout [38]. The IR filter was set to essentially block other wavelengths outside the NIR range while allowing light with 960 nm to pass through. The reflected light imaging principle was applied, which makes the device more compact with the light source attached to the camera. A laptop computer was utilized as the image processing unit (IPU) with Vds Vein display system 4.0 (open-source software) for processing the captured vein image. In image edge detection, this software allows for accurately capturing image in the pixel and checking color change degree of each pixel to distinguish the boundary. Based on the differences of each pixel grayscale, various objects within the image can have obvious edge distinguishing features in their boundaries. Additionally, a simple convolutional filter was used to detect the horizontal and vertical luminosity changes on the image, and the value of each point was calculated by the weighted average to determine the edge. Moreover, window technology in the medical image field, including window width and window center, were used to select the range of image values of interest, it is because various arm surface structures have different image values. When a certain surface structure was displayed, the window width and window position suitable for observing the target site can be chosen to obtain the best display. In the device operation, the vein images were clearly captured with an optimal working distance of 15–20 cm preventing direct skin contact and giving adequate space for the phlebotomist in performing venipuncture. The model device was attached to a transportable stand to free both hands of the user to perform cannulation. Figure 3 shows the details of the device design. As compared to 10 cm working distance, uneven power distribution was seen with “green color” on the left and “blue color” on the right areas, while, in 25 cm, working distance negative spots for power distribution with “white color” was noted, see Figure 4. The relationship between the working distance and power distribution was analyzed at 1.5 cm, 3.5 cm, and 5.5 cm CMOS Lens-LED configurations. The result showed an inversely proportional relationship, as the distance increases, the power decreases. Moreover, it was observed that, at 15 cm working distance with 10–14 mW, the power distribution using three LEDs was comparable to five LEDs and seven LEDs at 20 cm and 25 cm, respectively. On this regard, it is signified that three LEDs are sufficient to supply the required 10–14 mW power for vein imaging, see Figure 5. As to the required power strength of 10–14 mW, it was reached and showed equal distribution in different CMOS Lens-LED distances 1.5 cm, 3.5 cm, and 5.5 cm using three LEDs, contrary to five LEDs and seven LEDs that have higher power strength in 3.5 cm CMOS Lens-LED distance as compared to 1.5 cm and 5.5 cm, with results shown in Figure 6a–c. Moreover, the variations in power distribution values with different LED-LED distances, such as 1 cm, 2 cm, 3 cm, and 4 cm, were analyzed and revealed less than 1% standard distribution (SD) values. The 10 cm working distance has the lowest SD value, but the power is 24 mW, which is far higher than 10–14 mW power required for the device. With this, the 15 cm working distance having 13.124 mW in 2 cm LED-LED distance was noted to be sufficient in consideration of the power strength that is shown in Figure 7.

### 2.2. Assessment of the NIR Vein Finder Prototype (Image Analysis)

A total of 242 subjects/participants were enrolled in the study in the actual in-vitro testing of the developed vein finder device. The subjects’ demographics, such as gender, age, height, and weight for the body mass index (BMI), including the skin tone, were considered as parameters, the same with the previous studies [5,11,13,22,31,38,39,40,41], and with the inclusion of arm circumference measurement by the researchers in this study to maximize the assessment process. Specifically, two sites were selected; the arm and dorsal hand for testing, because the subcutaneous veins in the said areas are commonly used for venous blood specimen collection for laboratory testing and intravenous (IV) route for therapy, respectively.

**Figure 8:**
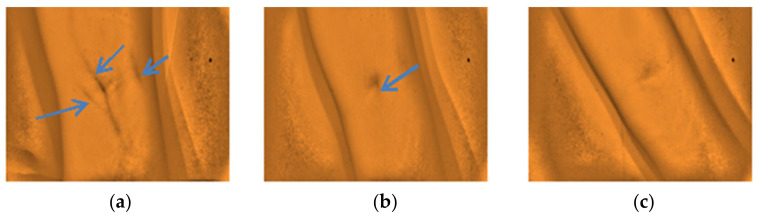
The types of captured images as results from the arm site. (**a**) Highly visible, (**b**) Visible, and (**c**) Non-visible using the developed vein finder.

**Figure 9:**
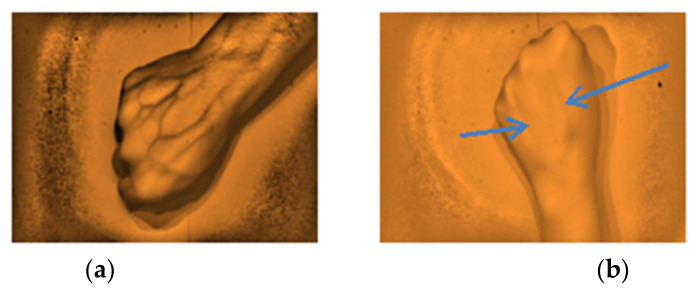
The types of captured images as results from the dorsal hand site. (**a**) Highly visible and (**b**) Visible using the developed vein finder.

The gathered images were evaluated by direct visual analysis performed by a practicing medical technologist and instructor of phlebotomy course in order to validate the capability of the developed vein finder. It was completed based on the criteria set by the researchers, as described in Figure 8 and Figure 9, and subjects’ age, body mass index (BMI), and skin tone/color.

**Figure 10:**
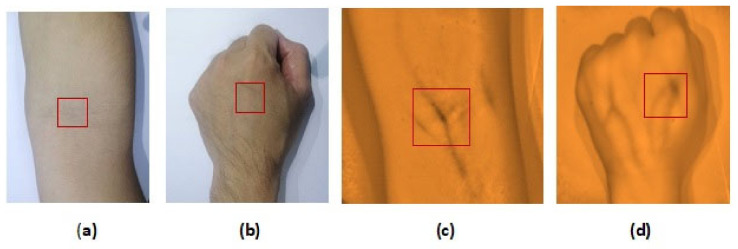
Peripheral subcutaneous vein imaging through visual approach (without the NIR veinfinder) (**a**) arm site (area: <1) and (**b**) dorsal hand site (area: <1) compared to the digital imaging approach (with the NIR vein finder) (**c**) arm site (area: 12865) and (**d**) dorsal hand site (area: 5112). The ImageJ software (National Institutes of Health, Bethesda, MD, USA) was used to quantify vein visibilities.

The captured subcutaneous vein images were classified based on the working definitions used in the study, (a) highly visible-the vein structure, as seen vividly in the arms of the subject and are enhanced using the vein finder, (b) visible-the veins’ image as seen after using the vein finder, and (c) non-visible-the veins’ image is not seen, even with the use of the device. In Figure 10, the difference between the captured vein images in the arm and dorsal hand sites using the two (2) approaches, visual and digital imaging approach, were quantitively compared while applying the ImageJ software, and the results showed a remarkable difference of area: <1 in both sites (visual approach), while a result of area: 12,865 and area: 5112 (digital imaging approach) in arm and dorsal hand sites, respectively. It indicates the improvement with regard to peripheral subcutaneous vein visibility using the developed vein finder.

The sample population were grouped according to age, as follows: Child 0–15, Young adult 16–30, Middle-aged adult 31–50, and Senior adult >50 [42], as to BMI which is defined as an estimate of the body fat content based on two factors, the height, and weight for calculation for both male and female were group into: Underweight <18.5, Normal 18.5–22.9, Overweight 23.0–24.9, and Obese ≥25.0 [43]. It is useful for this study to indicate the characteristic of being overweight and obesity. While for the skin color/tone classification: Very light complexion I, Light complexion II, Medium complexion III, Darker complexion IV, Dark complexion V, and Black complexion VI was used. This is based on the skin color/tone meter proposed by Thomas B. Fitzpatrick in 1975. It is compared on a person’s skin color and was used as a standard by healthcare professionals and aesthetic practitioners in the assessment of their patients [44]. 

### 2.3. Statistical Analysis

A study with a total of 242 human subjects was performed to evaluate the capability of the developed vein finder device, specifically for vein imaging and mapping in the arm and dorsal hand sites with consideration of gender, age, height, and weight for the body mass index (BMI), skin tone, and the arm circumference measurement as variables. All of the given variables were presented as frequency counts and in percentages that were utilized for the results analysis.

## 3. Results

### 3.1. Vein Visualization Rate with the Developed Vein Finder in the Arm and Dorsal Hand Sites

**Figure 11:**
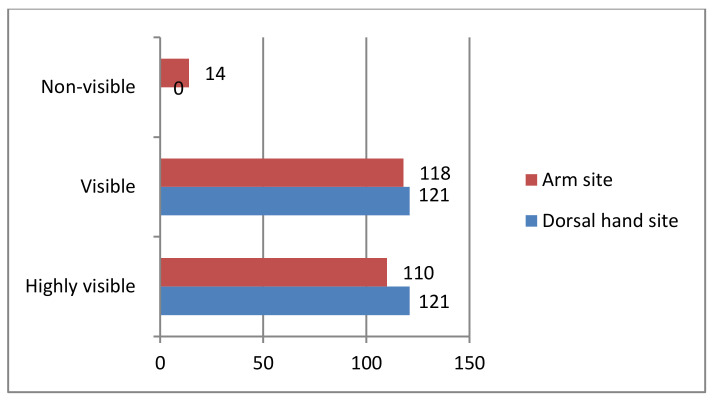
Vein visualization rate with the developed vein finder: 242 (100%) human subjects.

In the arm site, 110 (45.45%) are highly visible, 118 (48.76%) are visible, and 14 (5.79%) are non-visible, while, in the dorsal hand site, a result of 121 (50.00%) is equal for both highly visible and visible, while 0 (0.00%) for non-visible, as shown in Figure 11.

### 3.2. Assessment of the Vein Finder with the Different Parameters such as: Gender, Age, Body Mass Index (BMI), Skin Color/Tone, and Arm Circumference

These are the parameters that can be considered to be variables that may affect the capability of the vein finder to locate the subcutaneous veins in the site for cannulation.

#### 3.2.1. Arm Site: Results for Highly Visible and Visible Were Presented Based on the Given Parameters

There were 56 (63.64%) males out of 88 subjects and 54 (38.57%) out of 140 females which yielded highly visible results, while 32 (36.36%) males and 86 (61.43%) females yielded a visible classification presented in Table 1. This shows that, according to gender, males have vividly visible vein structures as compared to females. Moreover, the markedly increased visible result in female subjects suggests that the vein finder has the capability to make their subcutaneous arm veins visible.

Table 2 presents significantly increased number of visible results of six (60.00%) out of 10 subjects and 22 (61.00%) out of 36 subjects, in the child and middle-aged adult group, respectively. This shows that, according to age, the vein finder was able to improve visibility of veins in the arm site in child and middle-aged adult group while, higher results for highly visible were seen in young adult with 87 (50.28%) out of 173 and senior adult classification with 5 (55.55%) out of nine.

Table 3 presents a significantly increased number of visible results of 55 (67.07%) out of 82 subjects in obese classification. This indicates that according to BMI the device was able to improve subcutaneous vein visibility in the arm site even in human subjects with increased BMI scores. While, as expected, a highly visible vein result of 47 (63.51%) out of 74 was seen on subjects with normal BMI score.

Table 4 presents a significantly increased number of visible results of 68 (51.13%) out of 133 and five (100.00%) out of five in darker and dark complexion, respectively. This shows that according to skin color/tone classification, the vein finder was able to improve subcutaneous vein visibility even in human subjects with increased skin pigmentation. Additionally, it was noted that the device was able to improve visibility even in human subjects with light complexion, showing a result of four (66.67%) visible out of six.

Table 5 presents significantly increased number of visible results of 8 (80.00%) out of 10 and 46 (51.69%) out of 89 in human subjects with 34–30 cm and 29–25 cm arm circumference, respectively. This specifies that, according to the given classification, the device was able to improve subcutaneous vein visibility in human subjects, even with larger arm circumference size.

#### 3.2.2. Arm Site: Results for Non-Visible Were Presented Based on the Given Parameters

There was a total of 14 (5.79%) out of 242 subjects observed with non-visible peripheral subcutaneous veins. Table 6 shows the 14 subjects with their results based on the parameters (gender, age, BMI, skin tone, and arm circumference), which are considered to be potential confounders for the non-visibility of their veins in the arm site. With the 14 subjects, as to gender, the majority are female, eight (57.14%), as to age, many are in the young adult group, eight (57.14%), under obese classification, there are eight (57.14%), with darker complexion seven (50.00%), while seven (50.00%) are within the 25–29 cm range group for arm circumference, and some arms of the subjects were observed with stretch marks, three (21.43%), and skin marks, two (14.29%).

**Figure 12:**
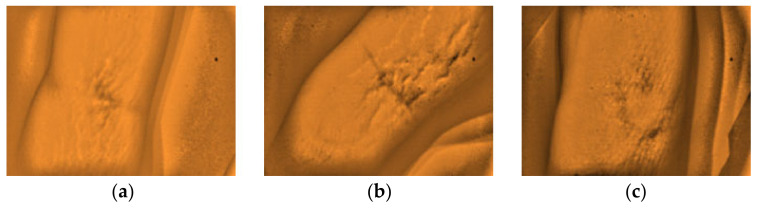
Arm images from three different subjects with stretch marks with non-visible vein (**a**–**c**).

**Figure 13:**
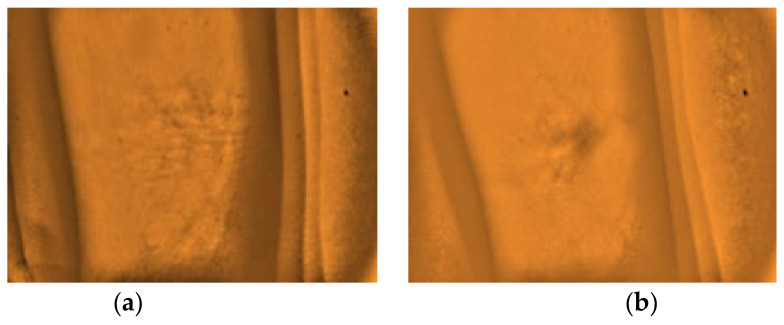
Arm images of two different subjects with skin marks due to a highly pigmented spot-epidermal melanin with non-visible vein (**a**,**b**).

**Figure 14:**
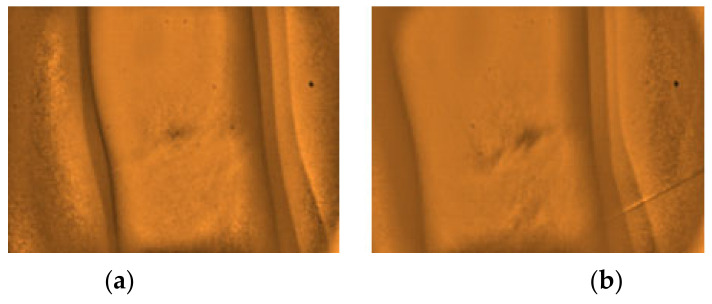
Arm images of two different subjects BMI = O, darker complexion (IV) skin tone with non-visible vein (**a**,**b**).

**Figure 15:**
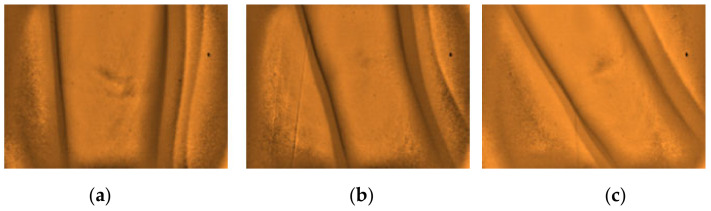
Arm images of three different subjects with BMI = N with non-visible vein (**a**–**c**).

**Figure 16:**
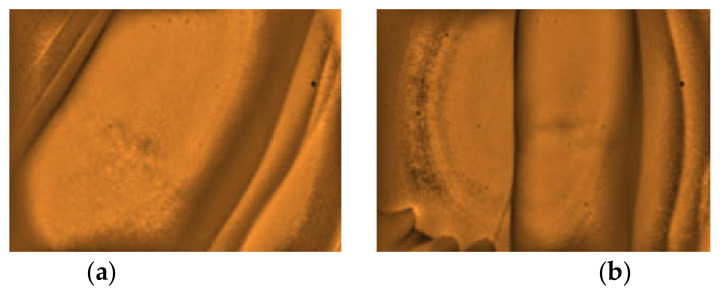
Arm images of two different child subjects with non-visible vein (**a**,**b**).

**Figure 17:**
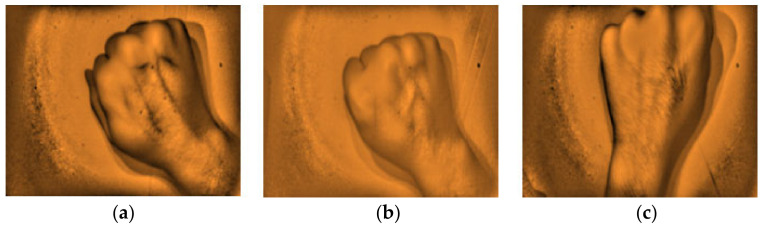
Dorsal hand images of three different subjects with hair structures with highly visible veins (**a**–**c**).

Furthermore, the researchers would like to report other observations that were noted from the analysis of the captured images with the non-visible results. The arms with the presence of stretch marks, highly pigmented spot with epidermal melanin [5], and from obese (BMI) subjects with darker complexion (Type IV) skin tone affects the visibility of the peripheral veins on the site, as presented in Figure 12, Figure 13 and Figure 14, respectively. Additionally, a non-visibility from subjects with normal BMI can be attributed to the depth of the vein that is more than 5 mm [29], and in child age group, due to the tiny vein structure with the presence of thicker subcutaneous tissues [22]. Figure 15 and Figure 16 present the said findings. In contrary, in Figure 17 as observed by the researchers, it is worth to mention that, even in the presence of hair structures in the dorsal hands of the three subjects, the imaging device was still capable of clearly mapping the veins, it is able to prevent the formation of a reflectance glare that can severely impair the vein contrast [41].

#### 3.2.3. Dorsal Hand Site: Results for Highly Visible and Visible Subcutaneous Veins Were Presented Based on the Given Parameters

There were 57 (61.29%) out of 93 male subjects and 64 (42.95%) out of 149 female subjects with highly visible results, while 36 (38.71%) male and 85 (57.05%) female subjects in visible classification, as presented in Table 7. This shows that according to gender, males have vividly visible vein structures compared to females. Moreover, the markedly increased visible results in female subjects suggest that the vein finder is capable of making the dorsal hand veins image visible.

Table 8 presents significantly increased visibility results of 10 (83.33%) out of 12 child subjects, 21 (53.85%) out of 39 middle aged adult, and six (60.00%) out of 10 senior adult. This shows that, according to age, the device was able to improve visibility of veins in the dorsal hand among child, middle aged adult, and senior adult groups. While a higher result for highly visible was evident among young adult with 97 (53.59%) out of 181.

Table 9 presents significantly increased visibility results of 16 (66.67%) out of 24 underweight subjects and 50 (55.56%) out of 90 obese subjects. This indicates that, according to BMI, the vein finder was able to improve subcutaneous vein visibility in human subjects with below normal and even with increased BMI scores. While, higher results for highly visible were noted in normal and overweight BMI classifications, with 47 (61.84%) out of 76 and 24 (60.00%) out of 40, respectively.

Table 10 presents significantly increased visibility results of 69 (49.64%) out of 139 and 4 (66.67%) out of 6 both in darker and dark complexion, respectively. This indicates that, according to skin color/tone classification, the vein finder was able to improve dorsal hand vein visibility, even in human subjects with increased skin pigmentation. Additionally, it is noted that the device was able to improve visibility in human subjects with light complexion, showing a result of four (66.67%) visible out of six.

## 4. Discussion

In general, based on the performed clinical trials, the near infrared imaging system of the developed vein finder in this study has shown its capability as an efficient guiding device through real-time vein pattern recognition, for both dorsal hand and arm sites. The reliable NIR color spectrum and wavelength of 960 nm was used, and noted with increased optical absorption of hemoglobin, resulting in an image of a darker vein with lighter skin tissue background. Moreover, NIR was known to identify images of lesions with distinct appearances, like melanoma, basal cell and squamous cell carcinoma, microvascular and inflammatory lesions, dermatophytes, verrucae, etc. [32]. This augmented subcutaneous vein imaging study using the develop vein finder device was performed in a well lightened and ventilated room like a patient’s room. Additionally, to maximize the assessment process, before the use of our vein finder, the researchers first investigated the veins on the target site, if it is visible or invisible with the naked eye. A total of 242 human subjects with variations as to gender, age, BMI, skin color/tone, and arm circumference were enrolled in the study. This is to validate the performance of the device by analyzing the captured vein images from the arm and dorsal hand sites. The intensity of the improved captured subcutaneous vein structures was compared through the use of the ImageJ software, as shown in Figure 10. The difference between the two (2) approaches, visual and digital imaging approach were quantitively compared, showing a remarkable improvement in the vein visibility, both in the arm and dorsal hand sites.

The vascular images that were captured by the vein finder prototype in the highly visible category showed a remarkable intensity ratio between the veins and the skin tissue background. While the 14 (5.79%) non-visible results, which were considered as the limitation of this device in the arm site will be thoroughly explained in the latter part of the discussion. The researchers would like to emphasize that the visible results, as defined in this paper, to be the human subjects with veins’ image only seen after using the vein finder, can be considered as the strength of this device, resulting in almost half of the subject population.

### 4.1. Based on Parameters: Both Arm and Dorsal Hand Sites (for Highly Visible and Visible)

Subcutaneous vein imaging can be challenged by different factors stated earlier. In consideration of those factors, the peripheral subcutaneous vein images were captured and analyzed while using the device. Having the results of increased highly visible among the males in both sites, these are associated with the observed physical visible vein structure which is larger compared to the veins of female subjects. The higher visible results in female subjects, also in both sites suggest that the device was able to capture vein images given the condition of having non-visible superficial veins observed with the naked eye. The device was able to improve the visibility of veins in child and middle-aged adult groups in both arm and dorsal hand sites. Although, subcutaneous fat and tiny veins of the young children are known factors that can affect vein visibility [22]. It makes venous access difficult to establish when compared to adults, particularly, among children younger than 3 years [13]. However, among senior adult group, vein visibility was also improved, as it was expected that vein size increases as we age, which is associated with the loss of elasticity in venous wall [45]. BMI as a parameter was considered in this study in accordance with previous studies relating to obesity as a factor, which may affect the visibility of the superficial veins [5,11,21,31,38], even stating that difficulty in cannulation and venipuncture increases with the BMI of obese to morbidly obese range [46]. With regard to our results, the device was able to improve the vein visibility of more than half of the population under the obese range. The skin color is another consideration for the assessment. The studies show that skin pigments, primarily melanin, affect the vein’s visibility [5,21,22,31,38,39,40,41]. It may cause challenges in locating the vein from individuals and races with darker skin tone. In this criterion, our device was able to improve the vein image of all the subjects with dark complexion in the arm site and more than fifty percent in the dorsal hand site.

The researchers included another parameter in order to maximize the assessment process. It is the measurement of the arm circumference in centimeter (cm). This is to observe the relationship between the arm size and the vein visibility. The results showed that the arm circumference size is inversely proportional to the visibility of the peripheral arm veins. Additionally, the result revealed that the device was capable of improving the veins visibility even with subjects having 25 to 34 cm arm circumference. 

### 4.2. Results of Non-Visible in the Arm Site

There are 14 (5.79%) out of 242 subjects with non-visible peripheral subcutaneous veins, as shown in Table 9. Analysis of the results showed that as to gender, majority are female; as to age, many are in the young adult group, for BMI more than half of them are in obese category. With regard to skin tone, majority are in III and IV classification and one V. Finally, for the arm circumference, the majority is in the 25–29 cm group.

The result of the prototype testing on vein visibility of 94.21% (arm site) and 100% (dorsal hand site) as compared to the visibility of the vein without the device having 54.55% (arm site) and 50.00% (dorsal hand site), in consideration of all the parameters used and with the minimal component of the device, showed that the researchers were able to meet the aim in developing a low-cost real-time vein finder.

The capability of the developed NIR vein finder device was further shown through a comparative analysis between the gathered data from the experimentation that was performed with the 242 human subjects and the commercial vein finder devices, such as the AccuVein (AccuVein Inc.), VeinViewer Flex (Christie Medical Holdings Inc.), and VascuLuminator (De Koningh Medical Systems). The result of the analysis shows the advantages of the prototype based on the following: minimal used components, low-cost, reproducibility, and with its reliability rate in vein mapping/viewing as compared to commercial devices, see Table 11.

## 5. Conclusions

In the study, it was presented that the developed simple vein finder device using NIR technology with low-energy consumption reflected light principle is highly efficient for real-time peripheral subcutaneous vein imaging. It is based on the analysis of the captured images real-time, using the naked eye as compared with the use of the device, on the arm and dorsal hand sites of 242 human subjects with varying gender, age, BMI, skin color/tone, and arm circumference. The results showed a remarkable increase in the vein visibility in general without the use of a tourniquet. The capability of the developed vein finder can be considered to be a useful guiding device in locating the vein for cannulation, providing venous access for blood sampling, therapy, and other medical purposes at a very low cost when compared to the commercially available vein finders. The developed vein finder can also be used by medical practitioners and students as an instructional tool for their training.

## Figures and Tables

**Table 1:** Highly visible and visible according to gender.

	No. of Subjects	Highly Visible	Visible
Male	88	56	32
Female	140	54	86
TOTAL	228	110	118

**Table 2:** Highly visible and visible according to age.

		No. of Subjects	Highly Visible	Visible
Child	0–15	10	4	6
Young adult	16–30	173	87	86
Middle-aged adult	31–50	36	14	22
Senior adult	>50	9	5	4
TOTAL		228	110	118

**Table 3:** Highly visible and visible according to BMI.

		No. of Subjects	Highly Visible	Visible
Underweight	<18.5	23	12	11
Normal	18.5–22.9	74	47	27
Overweight	23.0–24.9	39	20	19
Obese	≥25.0	82	27	55
TOTAL		218	106	112

**Table 4:** Highly visible and visible according to skin color/tone.

		No. of Subjects	Highly Visible	Visible
Light complexion	II	6	2	4
Medium complexion	III	84	43	41
Darker complexion	IV	133	65	68
Dark complexion	V	5	0	5
TOTAL		228	110	118

**Table 5:** Highly visible and visible according to arm circumference.

In Centimeter (cm)	No. of Subjects	Highly Visible	Visible
30–34	10	2	8
25–29	89	43	46
20–24	123	62	61
15–19	6	3	3
TOTAL	228	110	118

**Table 6:** Human subjects 14 (5.79%) with Non-visible subcutaneous veins.

Ref No.	Gender	Age	BMI	Skin Tone	Arm Circumference	Remarks
011	F	20	O	III	29	Stretch marks
046	M	21	O	IV	26	Obese, Darker complexion
058	M	19	O	IV	29	Skin marks
060	F	19	O	IV	27	Skin marks
069	M	19	O	IV	31	Obese, Darker complexion
095	M	50	O	IV	33	Obese, Medium complexion
112	F	18	N	III	24	Tiny/deep vein *
125	F	34	O	IV	26	Obese, Darker complexion
131	F	21	UW	III	21	Tiny/deep vein *
228	F	20	N	III	21	Tiny/deep vein *
232	M	39	O	IV	29	Stretch marks
233	M	67	OW	V	26	Stretch marks
241	F	13	NA	III	23	Child, Tiny/deep vein *
245	F	2	NA	III	15	Child, Tiny/deep vein *

**Legend:** M—Male; F—Female; BMI—Body Mass Index; N—Normal; UW—Underweight; OW—Overweight; O—Obese; NA—Non-applicable. * See discussion of results for non-visible.

**Table 7:** Highly visible and visible according to gender.

	No. of Subjects	Highly Visible	Visible
Male	93	57	36
Female	149	64	85
TOTAL	242	121	121

**Table 8:** Highly visible and visible according to age.

		No. of Subjects	Highly Visible	Visible
Child	0–15	12	2	10
Young adult	16–30	181	97	84
Middle-aged adult	31–50	39	18	21
Senior adult	>50	10	4	6
TOTAL		242	121	121

**Table 9:** Highly visible and visible according to BMI.

		No. of Subjects	Highly Visible	Visible
Underweight	<18.5	24	8	16
Normal	18.5–22.9	76	47	29
Overweight	23–24.9	40	24	16
Obese	≥25	90	40	50
TOTAL		230	119	111

**Table 10:** Highly visible and visible according to skin color/tone.

		No. of Subjects	Highly Visible	Visible
Light complexion	II	6	2	4
Medium complexion	III	91	47	44
Darker complexion	IV	139	70	69
Dark complexion	V	6	2	4
TOTAL		242	121	121

**Table 11:** Comparative analysis between the developed vein finder device and commercial vein finder devices with NIR technology.

Parameters	Developed Vein Finder Device	Commercial Vein Finder Devices
1. Minimal used components (for the vein finder prototype)	a. 3 NIR LED (Fongsam) 960 nm b. IR (IMX238 Sony) CMOS camera with IR filter c. Free software and laptop as the image processing unit d. Power consumption: 4.5 V e. Size: 7 × 9 × 10 cm f. Working distance: 15–20 cm g. Weight: 0.4 kg	1. AccuVein (AccuVein Inc.) a. two safe barcode-scanner class lasers: an invisible infrared and a visible red b. Dual lens [47] c. Projector system [48] d. Power consumption: 3.6 V, 180 min lasting, 3100 mAh e. Size: 5 × 6 × 20 cm f. Working distance: 10 to 45 cm g. Weight: 0.275 kg [47] 2. VeinViewer Flex (Christie Medical Holdings Inc.) a. NIR light (Minimum of 5 lumens) b. HD imaging and exclusive Df2 (digital full field) technology [49] c. Projector system [48] d. Power consumption: Fast-swap lithium ion batteries or AC (outlet) Up to 2 h continuous run time per battery e. Size: 29.464 × 10.16 × 4.318 cm f. Working distance: 30 cm g. Weight: 0.7 kg [49] 3. VascuLuminator (DKMP) a. LED, SFH 4235, (Osram, Munich, Germany) 850 nm [22] b. IR CCD camera with VGA resolution (640 × 480) and adjustable focus lens with IR filter. [22] c. Monitor LCD system [48] d. Power consumption: DKMS bv charger type 330–00 at least 8 h e. Size: 54 × 48 × 170 cm (when assembled) [50] f. Working distance: ∼20 cm [22] g. Weight: 23 kg [50]
**2. Estimated cost in USD** **(Highly reproducible based on its production cost)**	80–100	4000 to 7000 [36]
**3. Reliability Rate (%)** **a. Human in-vitro testing**	Arm site: 94.21% Dorsal hand site: 100.00% *of the 242 human subjects*	93.00% [35]

## Data Availability

Data sharing is not applicable to this article.
